# Influence of Leaders’ Emotional Labor and Its Perceived Appropriateness on Employees’ Emotional Labor

**DOI:** 10.3390/bs14050413

**Published:** 2024-05-15

**Authors:** Xiuli Tang, Yingkang Gu

**Affiliations:** 1 School of Event and Communication, Shanghai University of International Business and Economics, Shanghai 201620, China; 7972@suibe.edu.cn; 2 School of Economics and Management, Shanghai Polytechnic University, Shanghai 201209, China

**Keywords:** leaders’ emotional labor, employees’ emotional labor, perceived appropriateness, EASI, experimental research

## Abstract

Emotional labor is a crucial yet often overlooked aspect of effective leadership. To address this, the current study adopts the Emotion as Social Information (EASI) model as a theoretical framework to investigate the influence of leaders’ emotional labor and perceived appropriateness on employees’ emotional labor. A two (leaders’ emotional labor strategies: surface acting vs. deep acting) by two (perceived appropriateness: appropriate vs. inappropriate) between-subjects experiment was designed with a sample of 120 front-line service employees from hotels in Shanghai. The results showed that regardless of whether the perception of a leader’s surface acting was deemed appropriate or not, employees tended to perform surface acting, while the impact of the perceived appropriateness regarding the leader’s deep acting was different, wherein an appropriate display of deep acting by the leader significantly influenced employees to engage in deep acting themselves. The managerial implications and limitations of the findings are also discussed.

## 1. Introduction

Due to the growth of the service economy and the increased competition in service sectors, emotional labor has received close attention and generated a large amount of research. Emotional labor, defined as emotion-regulating and emotion-displaying behavior during interpersonal interactions in service-oriented workplaces, is an emotional transmission process containing an emotional contagion [1,2], which occurs not only between employees and customers, manifesting as the employees’ emotional labor, but also exists in interactions between leaders and employees, manifesting as the leaders’ emotional labor. Studies on leadership and emotional labor have verified that leaders also perform emotional labor in the process their of interactions with employees [3,4,5]. Leaders require more judgement than employees when it comes to effective leadership through emotional labor [6], in order to assess the appropriateness of their emotional labor strategies, and to effectively harness the function of positive emotions, thereby optimizing the performance of organizational members and, in turn, facilitating the overall performance of the organization. Consequently, it is interesting to explore how leaders’ emotional labor impacts employees’ attitudes and behaviors, which has the potential to provide a new understanding of how emotional labor can improve leadership in the workplace.

Humphrey (2008) puts forward the first leader emotional labor model by integrating the former research on emotional labor and leadership and holds that leaders’ emotional labor is a process whereby the leader uses emotional expression strategies to influence their subordinates, including surface acting and deep acting [6]. In general, leaders who smile more are perceived more positively by employees [7]. Leaders’ emotional expression through facial expression, intonation, and body language can be perceived by employees, which will affect employees’ attitude, behaviors [8] and performance [9,10]. However, it is essential to note that a leader’s emotional labor does not always yield favorable leadership outcomes. Existing studies have demonstrated that when leaders engage in apparent surface acting, they can evoke feelings of insincerity and manipulation among employees [4]. Consequently, employees react negatively to leaders’ apparent emotional displays. The above research indicates that employees’ perception of a leader’s emotional labor may affect its effectiveness. The impact of leaders’ emotional labor on employees’ emotional labor in the field of service management remains unknown. This is an important question regarding the leadership process and the interaction between leaders and employees. Any advice or recommendations on leaders’ emotional labor would be incomplete or inaccurate if they only considered the impact on employees without considering their perceptions. Therefore, this study aims to fill this gap by exploring how employees’ perception of their leaders’ emotional labor influences their own behaviors, specifically in terms of emotional labor.

We focus on the impact of leaders’ emotional labor on employees’ emotional labor as a work behavior in the field of organizational management for three reasons. Firstly, service contexts are highly emotional environments [11]. The emotional labor of front-line service employees is crucial for maintaining service quality, customer satisfaction, and corporate profits [12,13], making all factors influencing employees’ emotional labor worthy of study. Secondly, leaders have a significant impact on their employees; thus, leaders’ emotional labor can serve as a role model to motivate employees’ emotional labor, leading to a positive “Trickle-down” effect [14]. Lastly, in terms of theoretical contributions, this study can enhance the understanding of leaders’ emotional labor in leadership and, more importantly, provide evidence on how emotional labor can facilitate effective leadership in the workplace. It can also partially unlock the ‘black box’ of the process by which leaders’ emotional labor affects employees’ emotional labor, and provide useful guidance on the appropriate use of emotion in leadership research.

As this study measures employees’ perceptions of leaders’ emotional labor, we applied the Emotion as Social Information (EASI) model. Emotions serve as a form of social information with functions that affect both the individual and those around them. The EASI model has good explanatory power in investigating the influencing mechanism of leaders’ emotional labor [1,15], and experimental research methods can effectively test the causal relationships between variables. Moreover, many scholars have used experimental approaches to study emotional labor, so this study will continue in this way. Hotels, as typical service enterprises with high customer contact, are deeply influenced by the “customer-centric” service philosophy. Regardless of their own emotional states, employees are required to provide a smile with service in accordance with organizational rules, making emotional labor an integrated part of hotel employees’ daily work. In organizational management practice, the frequent interactions between hotel leaders and employees mean that leaders’ management of their own emotions undoubtedly affects employees’ emotional labor. Therefore, the hotel service management context is a fruitful research scenario. Based on the EASI model, this study therefore adopted a two (leaders’ emotional labor: surface acting vs. deep acting) by two (perceived appropriateness: appropriate vs. inappropriate) between-subjects experimental design to explore the influencing mechanism of leaders’ emotional labor on employees’ emotional labor.

## 2. Literature Review and Hypotheses

### 2.1. Leaders’ Emotional Labor

Hochschild (1983) first proposed the concept of emotional labor [16], which was later defined by Grandey (2000) as emotional regulation, consisting of surface acting and deep acting strategies [17]. After Humphrey (2008) put forward the first leaders’ emotional labor model, Gardner et al. (2009) developed a more comprehensive model that includes three strategies: surface acting, deep acting, and genuine emotions. They also suggested that a leader’s emotional labor influenced follower trust and their perceived authenticity through cognitive and affective processes [18]. In line with organizational culture, many companies have established specific guidelines for interpersonal interactions among colleagues [19]. Therefore, leaders’ emotional labor aligns with the characteristics of emotional labor, occurring during interpersonal interactions, following prescribed emotional display rules, and impacting others. The leaders’ emotional labor is part of the research area of emotional labor.

Based on the above studies, this study defines leaders’ emotional labor as the complete process by which leaders regulate their feelings and emotional expressions to interact with and influence employees following certain rules. The emotional regulation strategy includes both surface acting and deep acting. Surface acting refers to when leaders only modify their visible emotional expressions without changing their inner feelings, while deep acting involves genuinely altering their inner emotions to align with their external expressions. In comparison to employees, leaders not only need to use different emotional labor strategies, but they also have to make more significant judgments when deciding on the most effective emotional labor approach to use within various workplace contexts for managing employees [6].

### 2.2. Emotions as Social Information (EASI)

In researching emotional transmission, the Affective Events Theory and the Emotional Contagion Theory are commonly used explanatory mechanisms. However, they are not always effective in explaining the relationship between leaders’ emotions and employees’ behaviors. This limitation has become more apparent as organizations increasingly recognize the positive functions of leaders’ negative emotions. To address this limitation, van Kleef (2009) proposed the Emotion as Social Information (EASI) model [1], based on the Feelings-as-information Theory [20] and the Two-system Model [21].

According to the EASI model, the impact of leaders’ emotions on employees’ behaviors depends on how employees process the emotional information they observe from their leaders. This processing involves two mechanisms: affective reactions and inferential processes. The affective reactions in the EASI model involve the process of emotional contagion, where employees consciously or unconsciously catch or share their leaders’ emotions. This leads to interpersonal effects at an emotional level. On the other hand, the inferential processes in the model involve the reception and processing of leaders’ emotional expressions. This results in interpersonal effects at a cognitive level. Both affective reactions and inferential processes can occur simultaneously and influence employees’ behaviors [1]. The extent of this influence depends on the level of informational processing and the perceived appropriateness of the leaders’ emotional expressions [22]. The EASI model defines information processing as the extent to which employees fully and deeply process the emotional information communicated by leaders. This process is influenced by employees’ own motivations and capacities for informational processing. Perceived appropriateness, on the other hand, refers to employees’ judgment regarding the degree to which the leader’s emotional expressions match the current workplace contexts.

It is important to note that the same emotional expression by a leader may be considered appropriate in one situation but inappropriate in another. For instance, a leader’s expressions of anger may be perceived as appropriate in the context of a follower’s low effort or suboptimal performance, but as rather inappropriate in the context of a follower’s high effort or adequate performance. Leaders’ emotional expressions often convey their evaluation of employees’ task completion statuses [23,24,25]. For example, when leaders express anger, it suggests their dissatisfaction with the task’s completion. Employees then process this information, judge its appropriateness, and make decisions regarding their next actions. The emotional reactions that follow emotional expressions can be considered to be the outcomes of inferential processes [26]. Similarly, the process of perceiving appropriateness also involves a certain degree of information processing, which can be seen as another result of inferential processes. Therefore, this study will investigate how employees’ perceptions of the appropriateness of leaders’ emotional labor impacts their own emotional labor.

### 2.3. Perceived Appropriateness

Leaders have different ways to express positive emotions to employees through surface acting and deep acting, which aligns with their work role expectations. The effectiveness of leaders’ emotional labor is directly influenced by whether their emotional expressions are appropriate for the given situation. It is important to note that leaders’ positive emotions do not always elicit positive behaviors from employees. This depends on whether leaders convey the right emotions at the right time [27].

According to the EASI model, the predictive power of emotional responses versus inferential processes in relation to behaviors depends on whether employees perceive leaders’ emotional expressions to be appropriate or not [28]. The decision to rely on affective reactions or inferential processes for judging leaders’ emotional expressions varies from person to person. Yang and Li (2017) [28] discovered that leaders’ appropriate emotional expressions, such as deep acting, had a positive impact on the performance of high-efficiency employees. This impact was mediated by affective reactions since high-efficiency employees tended to have stronger intrinsic motivations and placed greater emphasis on their internal emotional experiences and satisfaction. On the other hand, low-efficiency employees, who were more driven by extrinsic motivations and were focused on external factors and their influences, tended to rely on inferential processes to interpret leaders’ emotional labors [28]. To summarize, affective reactions can be an effective means for leaders to motivate deep acting in high-effort employees, while inferential processes may be a potential driver for surface acting among low-effort employees. 

Leaders’ emotional expressions often reflect their evaluations of employees’ performances [23,24,25]. Therefore, whether leaders express camouflage emotions, such as feigned happiness through surface acting, or genuine emotions, such as real happiness through deep acting, low-effort employees infer from these expressions that the leader is satisfied with their performance and that there is no need for them to alter their work status. Instead, they can simply maintain surface acting, which requires minimal effort. Research has also found that an excessive level of positive emotions can lead to organizational members being overly optimistic and reducing their effort [29]. On the other hand, if leaders engage in apparent surface acting, feigning emotions such as happiness, high-effort employees perceive this to be insincere and manipulative [4]. This inappropriate emotional expression causes high-effort employees to develop aversions to leaders, leading to negative emotional effects and prompting employees to engage in surface acting. According to van Kleef (2016) and supported by previous research, when observers are also the targets of emotional expressions, some emotional expressions are more likely to be deemed inappropriate and can even elicit negative emotional reactions from observers [30]. Hence, the perceived appropriateness likely plays an important role in the influence of leaders’ surface acting on employees’ surface acting. Taken together, we propose the following hypotheses:

**H1a:**  *When leaders engage in surface acting, the perceived appropriateness of the leaders’ surface acting significantly influences employees’ surface acting. In particular, when leaders’ surface acting is perceived by employees as being inappropriate, employees are more likely to engage in higher levels of surface acting.*

**H1b:**  *When leaders engage in surface acting, the perceived appropriateness of this surface acting significantly influences employees’ deep acting.*

When leaders genuinely display emotions such as true happiness through deep acting, high-effort employees perceive them to be sincere and credible [29]. This appropriate emotional expression triggers positive emotional contagions and subsequently motivates high-effort employees to engage in deep acting at work. Per the EASI model, the intensity and authenticity of emotional expressions impact their perceived appropriateness to observers [1]. Cheshin et al. (2018) conducted a study and discovered that mild expressions by employees were perceived as being more sincere and appropriate to customers, compared to a display of strong joy or anger [31]. Additionally, genuine emotional expressions may not always be beneficial for effective leadership in certain situations [32]. Although both deep acting and surface acting involve conveying positive emotions, the level of genuine happiness displayed through deep acting is much stronger than the feigned happiness displayed through surface acting. However, for low-effort employees, leaders’ deep acting is more likely to be perceived as insincere or inappropriate, and it may not effectively inspire their own deep acting. Hence, the perceived appropriateness of the deep acting likely plays an important role in the influence of leaders’ deep acting on employees’ deep acting. Taken together, we propose the following hypotheses:

**H2a:**  *When leaders engage in deep acting, the perceived appropriateness of the deep acting to employees significantly influences the employees’ deep acting. In particular, when leaders’ deep acting is perceived as being appropriate, employees are more likely to engage in higher levels of deep acting.*

**H2b:**  *When leaders engage in deep acting, the perceived appropriateness of this to employees significantly influences employees’ surface acting*.

## 3. Research Design

### 3.1. Methodology

Emotional labor is dependent on the context in which it occurs. Therefore, exploring the impact of leaders’ emotional labor on employees’ emotional labor necessitates an examination within a specific organizational context. Previous studies on the relationship between the two have often been conducted using questionnaires, but this quantitative approach can only explore the correlation between variables. The advantage of an experimental research method lies in its ability to artificially control the experimental conditions in order to eliminate potential influences from other factors, and to effectively test the causal relationships between variables. Among these, a scenario-based experiment provides a practical and cost-effective method that utilizes stimuli such as texts, images, or videos, often accompanied by questionnaires or computer-based implementations.

Due to the numerous factors that influence leader–employee interactions in real workplace settings and the challenges of controlling experimental conditions, a scenario-based experiment method, using a text as the medium to initiate independent variables, is better suited to meeting the research requirements and ensuring good internal validity. This justifies the choice of this method for the current study. Furthermore, several previous studies have successfully employed this method to investigate emotional labor [1,28,33,34]. 

### 3.2. Participants and Design

A two (leaders’ emotional labor strategies: surface acting vs. deep acting) by two (perceived appropriateness: appropriate vs. inappropriate) between-subjects experimental design was adopted in this study. The experimental text for this study was developed as follows: firstly, a very common scenario for hotel employees in their daily work must be selected; secondly, this scenario should demonstrate the connotation of the leaders’ emotional labor and also showcase the behaviors associated with emotional labor, as described by the leaders’ emotional labor scale; thirdly, similar studies utilizing the text initiation procedure can be referenced. Finally, considering the above principles and supported by the relevant literature [27], the experimental text was developed.

Participants were full-time employees from several four- and five-star hotels in Shanghai, all of whom were local, Chinese, front-line service employees. Recruitment was carried out with the consent of the HR departments of these hotels, resulting in a final sample size of 120. In line with the principle of voluntary participation, the experiment was conducted collectively during the hotels’ regular staff training periods. Among the 120 participants, there were 34 males (*M_age_* = 26.84, *SD_age_* = 6.35) and 86 females (*M_age_* = 28.63, *SD_age_* = 6.62).

### 3.3. Procedure

At the beginning of the experiment, a unified instruction was used to inform the participants about the objectives while also emphasizing the principles of confidentiality and anonymity to alleviate any concerns they may have had. Participants were asked to remain calm and to imagine themselves in a scenario where they were an employee of a high star hotel and had worked there for several years. Next, they learned that they had just completed an important corporate annual convention reception project together with a team of coworkers. Although all team members had a collective responsibility for the project, the effort that each team member put into the project was different. Half of the participants read that, compared to the other team members, they had put more effort into the project. The other half of the participants read that they had put in less effort than the other team members. This manipulation of the participant’s contribution to the team’s performance was used to create differential perceptions of the appropriateness of the leader’s emotional labor.

Participants then learned that the outcome of the project was uncertain, and they also were unsure whether the leader was satisfied with their team’s performance. The next day, the leader came up to them personally to evaluate their contributions in the project. Half of the participants read that the leader walked into the room and informed them that the project had been successfully completed. Although the leader seemed satisfied and smiled, he failed to acknowledge the team’s efforts. Instead, the leader just briefly encouraged them to continue exerting more effort before turning away. Afterward, they started on their daily work. Meanwhile, the other half of the participants read that the leader arrived and joyfully announced that the project had been completed successfully. The leader appeared genuinely pleased and expressed sincere gratitude for the team’s efforts. He encouraged everyone to continue making further contributions. Even as the leader left, his face was still beaming with a smile. Then, they started on their daily work. 

The perceived appropriateness of the leader’s emotional labor depended on both the amount of effort the participant had put into the project and the leader’s emotional display (surface acting or deep acting). That is, the surface acting depicted by the leader would be rather inappropriate if the participant had put in more effort than the rest of the team, whereas the leader’s surface acting would be relatively appropriate in the condition in which the participant had put in less effort than the rest of the team. The other way around, the leader’s deep acting would be appropriate in the case where the participant had put in more effort, but relatively inappropriate in the case where the participant had put in less effort.

After participants read the scenario, we assessed their emotional labor using the widely used scale initially developed by Grandey [35]. This scale demonstrated good internal consistency and consisted of 11 items. Five items measured surface acting, such as “Facing customers, I would pretend to be in a good mood even if I was not”. Six items measured deep acting, such as such as “Facing customers, I look happy not only on the outside but feel happy on the inside”. In this study, the reliability of surface acting (Cronbach’s α) was 0.84, and for deep acting, it was 0.88.

Finally, we used two manipulation checks to assess whether participants had understood which emotional labor strategies the leader had employed, and whether they had put in more or less effort than the rest of their team. Both items were dichotomous choices between surface acting or deep acting, and between more or less effort, respectively. In addition, participants were asked to report the perceived appropriateness of the leader’s emotional display. They were given a single item where they had to choose between two dichotomous options: appropriate or inappropriate. The experimental data were valid if the perceived appropriateness reported by the participant was consistent with the assumed appropriateness of the experimental scenario, and vice versa.

## 4. Results

### 4.1. Manipulation Checks

Out of 120 participants, 7 participants (5.83%) failed to indicate which emotional labor (surface acting or deep acting) the leader had displayed, and 2 participants (1.67%) failed to indicate whether they had put in more or less effort than the rest of the team. The remaining 111 participants (92.5%) reported a perceived appropriateness that was consistent with the assumed appropriateness of the experimental scenario and valid for subsequent analysis. Among them, 35 participants (31.53%) reported that the leader’s surface acting was appropriate, 32 participants (28.83%) reported that the leader’s surface acting was inappropriate, 23 participants (20.72%) reported that the leader’s deep acting was appropriate, and 21 participants (18.92%) reported that the leader’s deep acting was inappropriate. This shows that the scenario was fully understood as intended by the participants and that the experimental manipulation was valid.

### 4.2. Hypotheses Tests

The main effect of the leader’s emotional labor on the employees’ surface acting was significant (*F*_(1,107)_ = 16.50, *p* = 0.000, *η*^2^ = 0.13), and the main effect of the leader’s acting’s perceived appropriateness on employees’ surface acting was not significant *(F*_(1,107)_ = 2.65, *p* = 0.11, *η*^2^ = 0.02), while the interaction effect was significant *(F*_(1,107)_ = 5.15, *p* = 0.03, *η*^2^ = 0.05). Meanwhile, the main effect of the leader’s emotional labor on employees’ deep acting was significant (*F*_(1,107)_ = 15.37, *p* = 0.000, *η*^2^ = 0.13), and the main effect of the leader’s acting’s perceived appropriateness on employees’ deep acting was not significant *(F*_(1,107)_ = 1.51, *p* = 0.22, *η*^2^ = 0.01), while the interaction effect was significant *(F*_(1,107)_ = 4.381, *p* = 0.04, *η*^2^ = 0.04). This indicates that the main effect of the leader’s emotional labor on the employees’ emotional labor is significant, while the main effect of the leader’s acting’s perceived appropriateness on employees’ emotional labor is not significant. 

**Figure 1:**
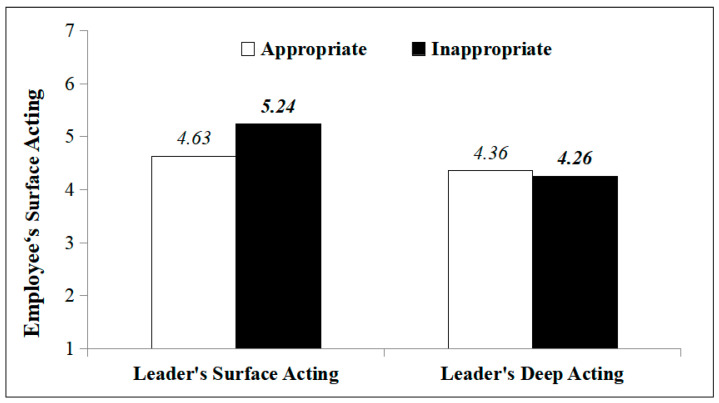
The influence of leaders’ emotional labor and its perceived appropriateness on employees’ surface acting.

Through simple effects analysis, this study found that when a leader engaged in surface acting, employees’ surface acting under the leader’s inappropriate surface acting (*M* = 5.24, *SD* = 0.64) was significantly higher than that under the leader’s appropriate surface acting *(M* = 4.63, *SD* = 0.82), *F*_(1,107)_ = 9.57, *p* = 0.003, *η*^2^ = 0.08. That is to say, when the leader engages in surface acting, the perceived appropriateness of the leaders’ surface acting has a significant influence on the employees’ engaging in surface acting, where the inappropriate surface acting of the leader triggered a higher amount of surface acting being performed by the employee. H1a was supported. When a leader engaged in deep acting, the employees’ surface acting had no significant difference in occurrence in accordance with whether the leader’s deep acting was appropriate (*M* = 4.26, *SD* = 0.74) or inappropriate *(M* = 4.36, *SD* = 0.99), *F*_(1,107)_ = 0.17, *p* = 0.68, *η*^2^ = 0.002. That is to say, when a leader engages in deep acting, the perceived appropriateness of the leaders’ deep acting has no significant influence on the employees’ performing surface acting. H2b was not supported, as shown in Figure 1.

Similarly, when a leader engaged in surface acting, there was no significant difference in the employees’ engagement in deep acting whether the surface acting of the leader was appropriate (*M* = 5.05, SD = 0.82) or inappropriate (*M* = 5.17, *SD* = 0.44), *F*_(1,107)_ = 0.47, *p* = 0.49, *η*^2^ = 0.004. That is to say, when a leader engages in surface acting, the perceived appropriateness of the leader’s deep acting has no significant influence on employees’ engaging in deep acting. H1b was not supported.

**Figure 2:**
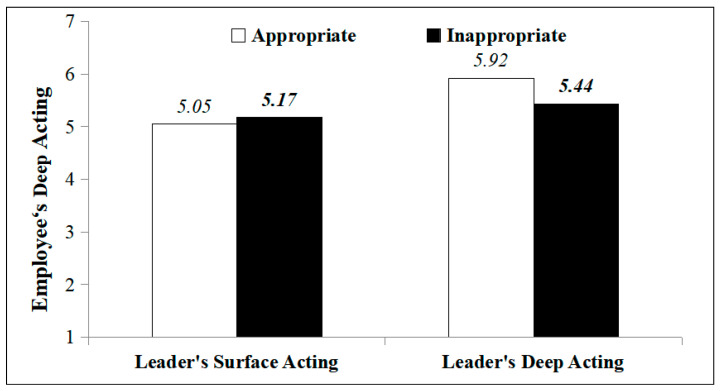
The influence of leaders’ emotional labor and its perceived appropriateness on employees’ deep acting.

When a leader engaged in deep acting, the employees’ deep acting following the leaders’ appropriate deep acting (*M* = 5.92, *SD* = 0.76) was significantly higher in occurrence than that following the leaders’ inappropriate deep acting (*M* = 5.44, *SD* = 0.98), *F*_(1,107)_ = 4.57, *p* = 0.04, *η*^2^ = 0.04. That is to say, when a leader engages in deep acting, the perceived appropriateness of the leaders’ deep acting has a significant influence on employees’ engaging in deep acting, where the appropriate deep acting of the leader triggers a higher amount of deep acting from the employee. H2a was supported, as shown in Figure 2.

## 5. Conclusions and Discussion

### 5.1. Conclusions

Emotional labor constitutes an essential facet of the daily work of front-line employees in service-oriented enterprises [11], playing a pivotal role in the effective management and operation of such organizations. The effectiveness of leadership is often measured by the responses it elicits from subordinates, with the phenomenon of the trickle-down effect in leader–employee interaction being of particular concern. This study has revealed a profound influence of leaders’ emotional labor on that of their employees. When leaders engage in surface acting, it serves as a catalyst for employees to mirror this behavior in their own emotional labors. Similarly, when leaders exhibit deep acting, it encourages employees to adopt a similar approach. This suggests that a clear trickle-down effect is in operation, with the emotional labor exhibited by leaders having a direct bearing on the emotional labor of their employees. Specifically, it also revealed that whether the employees’ perceived leaders’ surface acting to be appropriate or not does not reduce employees’ tendency to engage in surface acting. In fact, when leaders’ surface acting is perceived as being inappropriate, it paradoxically leads to an increase in the employees’ engaging in surface acting. Conversely, the impact of the employees’ perceiving the appropriateness of the leaders’ deep acting differs from that of their perceptions of the leaders’ surface acting’s appropriateness. In cases where the leaders’ deep acting is perceived by employees as being appropriate, it serves as a powerful motivator for employees to engage in more deep acting in their own emotional labor, leading to a more harmonious and productive work environment. In conclusion, this study has illuminated the intricate dynamics of emotional labor in organizational leadership and its cascading effects on employees. It is clear that emotional labor is a strategic tool in the leadership toolkit that leaders must use with care and consideration, as its impact can be both profound and far-reaching. 

### 5.2. Theoretical Contributions

Firstly, this study deepens the understanding of leaders’ emotional labor. By exploring the impact of leaders’ emotional labor on employees’ analogous behaviors (employees’ emotional labor), this study enriches the understanding of and the scholarly findings on leaders’ emotional labor. The simultaneity of the service process and the customer consumption process gives employees’ emotional labor behaviors a degree of autonomy that is challenging for organizations to manage in real time. Although both the surface acting and the deep acting performed by leaders are strategic expressions of positive emotions conducive to triggering positive emotional experiences in employees [36,37,38], our findings indicate that the leaders’ emotional labor is not always effective when it comes to influencing employees’ analogous behaviors (employees’ emotional labor), and may even be counterproductive at times.

Secondly, this study enriches the evidence of how emotional labor can facilitate effective leadership in the workplace. Leadership effectiveness is a hot topic in the field of organizational management, with the degree of effectiveness being partly contingent on the leader’s utilization of emotions [39]. Hence, research on leaders’ emotional labor has gained momentum in the past decade [40]. How employees, as recipients of emotions, perceive and interpret their leaders’ feigned emotions (their surface acting and deep acting) is a decisive factor in the formation and development of the interpersonal relationships affected by feigned emotions [41]. As Koning and Van Kleef (2015) [27] pointed out in their research, it is not necessarily the leaders’ positive emotional expressions that foster subordinates’ positive behaviors, but rather whether leaders express the appropriate emotions at the right time. While authenticity is a key indicator of how employees perceive and evaluate their leaders [10], which is crucial for the development of leadership [41,42], leaders must also consider how employees judge which emotional expressions are appropriate in which contexts. The key task for leaders in utilizing emotional labor is to appropriately contextualize specific situations to effectively leverage the beneficial impact of positive emotions. Our research findings support and validate this point.

Lastly, this study validates and extends the application of the EASI model. Based on the EASI model’s assumption of an “interpersonal effect” at the emotional level between leaders and employees, this study confirms that leaders’ emotional labor indeed triggers analogous emotional labor being performed among employees. As predicted by the EASI model, a leader’s inappropriate surface acting leads to higher levels of employees engaging in surface acting, while a leader’s appropriate deep acting leads to employee intensely engaging in deep acting. The integration of this study with the theory of a leader’s emotional labor, coupled with the setting of local organizational contexts, also extends the application of the EASI model, offering implications for subsequent studies.

### 5.3. Management Insights

The essence of leadership is to influence others, and emotional expression is an important source of social influence [1]. The management of emotions between leaders and employees is no longer considered to be a non-core task that has nothing to do with productivity and job performance [43]. Just as employees in customer service increase customer loyalty by triggering positive emotions through emotional labor, the leaders’ emotional transmission during the process of emotional labor also affects employees’ emotional involvement and job performance. Therefore, the application of leaders’ emotional labor should be valued in order to establish positive superior–subordinate relationships.

On this basis, leaders should also correctly employ emotional labor strategies to achieve better leadership effectiveness. The appropriate transmission of positive emotions is a crucial catalyst for encouraging employees to engage in deep acting. This is because leaders’ genuine and appropriate transmission of positive emotions provides a form of a work resource and a psychological support for employees. It not only reduces the emotional exhaustion associated with emotional labor to a certain extent [44], but also stimulates employees’ enthusiasm for work. This is a positive and effective method for the motivation that managers seek, unlike traditional material incentives, and has far-reaching implications for management practices.

## 6. Limitations and Suggestions for Future Study

Despite these findings, this study has some limitations that can be refined in future studies. On the one hand, the participants were limited to front-line service employees from domestic hotels in China, so the applicability of the findings needs further validation. Future studies could consider the impact of cultural differences, including both macro-social and micro-organizational cultural differences. For instance, the shaping of individuals’ psychology and behavior by individualist and collectivist cultures may be different. Leaders’ emotional labor and employees’ perceptions of its appropriateness may also vary across cultural contexts. 

On the other hand, this study applied experimental scenario methods to an emotional labor study and identified certain experimental effects. However, there are some limitations that need to be explored in further studies. Firstly, the experimental scenarios designed in this study were hypothetical rather than within real organizational contexts. In real organizational settings, employees engage in numerous interpersonal interactions with both their direct and indirect supervisors as well as their colleagues, including the fit degree between the supervisors and subordinates [45], as well as their existing emotional foundations, all of which may influence employees’ emotional labor. Yang and Li (2017) conducted a public goods game experiment to manipulate the efficiency levels of employees and found that the impact of a leader’s emotional labor on an employee’s performance varied depending on the employee’s efficiency level [28]. However, the employee effort level in our research was simulated or imagined rather than real. Secondly, this study primarily focuses on the emotional labor within the experimental scenario and neglects the potential influence of participants’ pre-existing emotions regarding their perceptions of their leaders’ emotional labor, as both pre-existing and immediate emotions may have a certain impact on decision-making [46], which requires further investigation in future studies. Finally, this study used only a single experimental method to explore the role of the perceived behavior appropriateness, and should integrate additional research methods in the future, such as questionnaire surveys, to explore the role of factors such as motivation and attribution styles in influencing employees’ perceptions of behaviors’ appropriateness.

## Data Availability

The datasets analyzed in this study are available from the corresponding author upon reasonable request.

## References

[1] van Kleef G.A. (2009). How emotions regulate social life: The emotions as social information (EASI) model. Curr. Dir. Psychol. Sci..

[2] Zhang Q., Liu J., Yan Z., Chen C. (2016). The mechanism of emotional contagion. Acta Psychol. Sin..

[3] Brotheridge C.M., Grandey A.A. (2002). Emotional Labor and Burnout: Comparing Two Perspectives of “People Work”. J. Vocat. Behav..

[4] Fisk G.M., Friesen J.P. (2012). Perceptions of leader emotion regulation and LMX as predictors of followers’ job satisfaction and organizational citizenship behaviors. Leadersh. Q..

[5] Wang R., Kim S.J., Kwon I. (2023). The Profiles and Antecedents of Supervisor-Directed Emotional Labor Strategies: The Role of Self-Identity and LMX Orientations in Emotional Labor Strategy. Behav. Sci..

[6] Humphrey R.H., Pollack J.M., Hawver T. (2008). Leading with emotional labor. J. Manag. Psychol..

[7] Trichas S., Schyns B. (2012). The face of leadership: Perceiving leaders from facial expression. Leadersh. Q..

[8] Wang Z., Chen L. (2014). The Effects of Leaders’ Affect: An Integrative Perspective Based on Multi-level Theories. Adv. Psychol. Sci..

[9] Tang X., Gu Y., Cui L. (2017). Influence of Leader and Employee Emotional Labor on Service Performance: A Hierarchical Linear Modeling Approach. Soc. Behav. Personal..

[10] Moon T.W., Hur W., Choi Y.J. (2019). How leaders’ perceived emotional labor leads to followers’ job performance: A serial mediation model. J. Serv. Theor. Pract..

[11] Yang C., Chen Y., Zhao X. (2019). Emotional Labor: Scale Development and Validation in the Chinese Context. Front. Psychol..

[12] Groth M., Hennig-Thurau T., Walsh G. (2009). Customer reactions to emotional labor: The roles of employee acting strategies and customer detection accuracy. Acad. Manag. J..

[13] Hur W., Moon T., Jung Y.S. (2015). Customer response to employee emotional labor: The structural relationship between emotional labor, job satisfaction, and customer satisfaction. J. Serv. Mark..

[14] Wayne S., Hoobler J., Marinova S.V., Johnson M.M. (2008). Abusive behavior: Trickle-down effects beyond the dyad. Acad. Manag..

[15] Liu X., Fu J. (2022). The theory and application of the Emotions as Social Information (EASI) Model. Adv. Psychol. Sci..

[16] Hochschild A.R. (1983). The Managed Heart: Commercialization of Human Feeling.

[17] Grandey A.A. (2000). Emotional regulation in the workplace: A new way to conceptualize emotional labor. J. Occup. Health Psych..

[18] Gardner W.L., Fischer D., Hunt J.G.J. (2009). Emotional labor and leadership: A threat to authenticity?. Leadersh. Q..

[19] Carlson D., Ferguson M., Hunter E., Whitten D. (2012). Abusive supervision and work–family conflict: The path through emotional labor and burnout. Leadersh. Q..

[20] Schwarz N., Clore G.L. (1983). Mood, misattribution, and judgments of well-being: Informative and directive functions of affective states. J. Personal. Soc. Psychol..

[21] Kahneman D., Frederick S., Gilovich T., Griffin D., Kahneman D. (2002). Representativeness revisited: Attribute substitution in intuitive judgment. Heuristics and Biases: The Psychology of Intuitive Judgment.

[22] Van Kleef G.A., Homan A.C., Cheshin A. (2012). Emotional influence at work: Take it EASI. Organ. Psychol. Rev..

[23] de Melo C.M., Carnevale P.J., Read S.J., Gratch J. (2013). Reading people’s minds from emotion expressions in interdependent decision making. J. Pers. Soc. Psychol..

[24] Hareli S., Hess U. (2010). What emotional reactions can tell us about the nature of others: An appraisal perspective on person perception. Cogn. Emot..

[25] van Kleef G.A. (2014). Understanding the positive and negative effects of emotional expression in organization: EASI does it. Hum. Relat..

[26] Smith L.W., Rose R.L. (2020). Service with a smiley face: Emotional contagion in digitally mediated relationships. Int. J. Res. Mark..

[27] Koning L.F., Van Kleef G.A. (2015). How leaders’ emotional displays shape followers’ organizational citizenship behavior. Leadersh. Q..

[28] Yang C., Li J. (2017). Is Emotional Fit or Emotional Dissonance Better Able to Improve Employees’ Performance?—An Experimental Study Based on the Leader Emotional Labor. Manag. Rev..

[29] Sy T., Côté S., Saavedra R. (2005). The contagious leader: Impact of the leader’s mood on the mood of group members, group affective tone, and group processes. J. Appl. Psychol..

[30] van Kleef G.A. (2016). The Interpersonal Dynamics of Emotion: Toward an Integrative Theory of Emotions as Social Information.

[31] Cheshin A., Amit A., van Kleef G.A. (2018). The interpersonal effects of emotion intensity in customer service: Perceived appropriateness and authenticity of attendants’ emotional displays shape customer trust and satisfaction. Organ. Behav. Hum. Decis..

[32] Wang G., Seibert S.E. (2015). The impact of leader emotion display frequency on follower performance: Leader surface acting and mean emotion display as boundary conditions. Leadersh. Q..

[33] Feng R., Bi Y., Fu X., Wang J., Li M. (2020). The interpersonal effects of fake emotion and the way it works. Adv. Psychol. Sci..

[34] Ma S., Huang M. (2006). Emotional Labor: Surface Acting and Deep Acting, Which One is Better?. Acta Psychol. Sin..

[35] Grandey A.A. (2003). When the smile must go on: Surface acting and deep acting as determinants of emotional exhaustion and peer rated service delivery. Acad. Manag. J..

[36] Kelly J.R., Barsade S.G. (2001). Mood and emotions in small groups and work teams. Organ. Behav. Hum. Dec..

[37] Johnson S.K. (2008). I second that emotion: Effects of emotional contagion and affect at work on leader and follower outcomes. Leadersh. Q..

[38] Damen F., Van Knippenberg D., Van Knippenberg B. (2008). Leader Affective Displays and Attributions of Charisma: The Role of Arousal. J. Appl. Soc. Psychol..

[39] Feng J., Liu S. (2018). A Literature Review of Leader Affect. Foreign Econ. Manag..

[40] Shao B., Lin X., Duan J. (2022). How leader emotional labour is associated with creativity: A self-determination theory perspective. Appl. Psychol..

[41] Zhu Y., Long L., Liu W. (2023). Can leader gratitude expression improve employee followership behavior? The role of emotional expression authenticity. Acta Psychol. Sin..

[42] Edelman P.J., van Knippenberg D. (2017). Training leader emotion regulation and leadership effectiveness. J. Bus. Psychol..

[43] Peng Z., Wang H., Gu F. (2011). The effect emergent leader plays on group emotion and employee innovative behavior. Stud. Sci. Sci..

[44] Lu X., Sun J. (2016). When leader-member exchange increases emotional exhaustion? The role of belief in reciprocity and power distance orientation. Acta Psychol. Sin..

[45] Peng J., Wang Z., Hou N. (2019). Do leaders and followers see eye to eye? Leader-follower fit in the workplace. Adv. Psychol. Sci..

[46] Chen J., Wang W. (2013). A Review of the Emotion as Social Information Model. Psychol. Dev. Educ..

